# The Role of Cognitive Development and Strategic Task Tendencies in the Bilingual Advantage Controversy

**DOI:** 10.3389/fpsyg.2018.01790

**Published:** 2018-09-25

**Authors:** Esli Struys, Wouter Duyck, Evy Woumans

**Affiliations:** ^1^Centre for Linguistics, Vrije Universiteit Brussel, Brussels, Belgium; ^2^Brussels Institute for Applied Linguistics, Vrije Universiteit Brussel, Brussels, Belgium; ^3^Center for Neurosciences, Vrije Universiteit Brussel, Brussels, Belgium; ^4^Department of Experimental Psychology, Ghent University, Ghent, Belgium

**Keywords:** bilingualism, cognitive control, inhibition, speed-accuracy trade-off, choice strategy

## Abstract

Recent meta-analyses have indicated that the bilingual advantage in cognitive control is not clear-cut. So far, the literature has mainly focussed on behavioral differences and potential differences in strategic task tendencies between monolinguals and bilinguals have been left unexplored. In the present study, two groups of younger and older bilingual Dutch–French children were compared to monolingual controls on a Simon and flanker task. Beside the classical between-group comparison, we also investigated potential differences in strategy choices as indexed by the speed-accuracy trade-off. Whereas we did not find any evidence for an advantage for bilingual over monolingual children, only the bilinguals showed a significant speed-accuracy trade-off across tasks and age groups. Furthermore, in the younger bilingual group, the trade-off effect was only found in the Simon and not the flanker task. These findings suggest that differences in strategy choices can mask variations in performance between bilinguals and monolinguals, and therefore also provide inconsistent findings on the bilingual cognitive control advantage.

## Introduction

The bilingual advantage in cognitive control assumes that bilinguals outperform monolinguals in conflict tasks, such as the Simon or flanker, due to their continued practice in handling between-language competition (for a recent review, see Zhou and Krott, 2016). These tasks typically contain a mixture of non-conflict (i.e., congruent) and conflict (i.e., incongruent) trials. Performance is consistently slower or less accurate for the latter (for a review study on these effects, see Lu and Proctor, 1995). Despite the general label of an *advantage*, the reported benefits for bilinguals are actually quite diverse (Hilchey and Klein, 2011), and not very consistent across studies: sometimes, they show better performance only on incongruent trials, but not on congruent trials (e.g., Schroeder and Marian, 2012; Marzecova et al., 2013; Pelham and Abrams, 2014); at other times, they outperform monolinguals on overall performance (e.g., Costa et al., 2009; Kapa and Colombo, 2013; Morales et al., 2013). And yet, there are also studies showing a combination of both (Bialystok et al., 2004; Tao et al., 2011; Yang et al., 2011).

Besides the varying manifestation of effects, bilingual benefits have become highly controversial because of repeated failures to replicate this superior performance altogether (e.g., Paap et al., 2015; von Bastian et al., 2016; de Bruin and Della Sala, 2017; Paap, in press). This has even led to the assertion that there is no coherent evidence for a bilingual advantage in cognitive control (Paap and Greenberg, 2013). Still, the lack of significant differences between groups of monolingual and bilingual participants does not necessarily mean that bilinguals and monolingual process these cognitive tasks in exactly the same way. There is some evidence that the processes needed for bilingual language control are not the same as those required by monolinguals (e.g., Hernandez et al., 2001), and that these differences have behavioral implications (e.g., Abutalebi et al., 2012). Therefore, it is recommended to abandon the quest for bilingual advantages and instead to focus on the question as to why at least some (but not all) bilinguals tend to process cognitive control tasks differently (but not always better) than monolinguals.

One explanation for this could be related to developmental differences between monolinguals and bilinguals because bilingual *advantages* are not consistently present across the lifespan of a bilingual individual (see Bialystok, 2007). As suggested by Bialystok et al. (2004), it is plausible that enhanced performance on conflict tasks only manifests itself in early childhood when individuals have not yet reached peak performance on these tasks. This in contrast to young adulthood, when performance is at ceiling level and environmental factors have little or no room to increase the efficiency of the processes involved in cognitive control. However, age cannot be the only factor to explain contradictory findings, because even research with children has produced bilingual advantage null effects (see, for instance, Antón et al., 2014).

One other explanation as to why bilingual advantages in cognitive control have only been observed in some but certainly not all studies can be related to the strategic choices made by individuals to carry out these tasks. In any task that involves the registration of response times and accuracy, such as in the interference tasks used to test the bilingual advantage, participants can optimize either speed or accuracy, or any compromise between both. Such conscious or unconscious strategic tendencies will have an effect on performance and this phenomenon is referred to as the speed-accuracy trade-off (Meyer et al., 1988). A tendency for speed may decrease response times at the cost of accuracy rates, whereas a tendency for accuracy may lead to slower response times but higher accuracy rates. This trade-off has been widely tested across various cognitive domains (see, for instance, Mackay, 1982; Forster et al., 2003), and it has been observed in interference tasks, such as the Simon (e.g., Hilchey et al., 2011; Ivanoff et al., 2014; van Wouwe et al., 2014) and flanker task (e.g., Rinkenauer et al., 2004; Wylie et al., 2009; Uemura et al., 2013).

Most studies about bilingual effects on cognitive control only focus on speed but not on accuracy. In a highly critical review article on the bilingual advantage, Paap et al. (2014) report that only 12 out of the 24 reviewed studies found lower response times for bilinguals than monolinguals (Luk et al., 2011; Tao et al., 2011; Salvatierra and Rosselli, 2011; Yang et al., 2011; Abutalebi et al., 2012; de Abreu et al., 2012; Poarch and van Hell, 2012; Schroeder and Marian, 2012; Kapa and Colombo, 2013; Marzecova et al., 2013; Morales et al., 2013; Pelham and Abrams, 2014), while information about the accuracy data is not provided. A separate analysis on the accuracy data of these 24 studies reveals that only five mention a bilingual advantage in terms of accuracy (Tao et al., 2011; Yang et al., 2011; Marzecova et al., 2013; Morales et al., 2013; Gathercole et al., 2014). This logically implies that the speed and accuracy outcomes did not align in the other studies reporting a bilingual advantage in speed processing and it could also indicate the presence of a speed-accuracy trade-off. One reason why analyses on accuracy are often neglected is because errors are rare in young adults performing cognitive control tasks. Error rates on these tasks are much higher in populations of children under the age of 12 (Bunge et al., 2002), which makes this group perfectly suitable for investigating the developmental aspects of differences in the speed-accuracy trade-off between bilinguals and monolinguals. Moreover, some studies on bilingualism and cognitive control in children have found advantages in response times but not in accuracy (e.g., Martin-Rhee and Bialystok, 2008; Barac and Bialystok, 2012; Poarch and van Hell, 2012), again suggesting a potential speed-accuracy trade-off also in that age group.

### The Present Study

This study set out to determine to what extent differences in strategic tendencies toward speed or accuracy between bilinguals and monolinguals explain part of the ongoing controversy surrounding the existence of a bilingual control advantage. It is well-known that the presence of two language systems in the bilingual mind generates conflict at various levels of linguistic analysis (e.g., van Heuven et al., 2008; Moreno et al., 2010; Blanco-Elorrieta and Pylkkanen, 2016) and that bilinguals must develop strategies to cope with this conflict in order to suppress the non-target language system and to activate the target one (e.g., FrenckMestre and Pynte, 1997). It has been proposed that domain-general interference tasks (such as the flanker or Simon task) generate conflict that is solved by the same processes as those required for daily bilingual language usage (e.g., Coderre et al., 2016). Strategic choices are not only needed to resolve the conflict generated by the most complex trials, but also to decide how to increase performance on these interference tasks. In general, individuals may optimize either speed or accuracy, which means that they can show faster response times at the cost of higher error rates, or instead be more accurate at a slower pace.

We hypothesize that bilinguals may show different strategies relative to monolinguals, after daily exposure to language conflicts and the need for developing strategies to overcome such conflict. This hypothesis is based on a review of the literature on the bilingual advantage. While some have challenged its existence based on reaction time data (Paap et al., 2014), their case could even be more convincing when error rates or accuracy of processing is considered. In some cases, better performance for bilinguals is only observed when reaction times and not accuracy scores are taken into account. This may be indicative of a selective speed-accuracy trade-off only for bilinguals, suggesting that bilinguals opt for a clear speed strategy when carrying out interference tasks, and this strategic choice may go at the cost of accuracy.

Our study intended to investigate this by assessing the correlation between response time (lower = better) and accuracy rates (higher = better), possibly showing that faster processing is compensated by lower accuracy. Additionally, we aimed to examine to what extent this speed-accuracy trade-off was related to developmental differences in bilinguals’ cognitive control performance. Recent literature on the interaction between bilingualism and cognitive control seems to indicate that bilingual benefits are more frequently found in young children than in young adults, thereby highlighting potential developmental factors affecting this interaction (for a recent review, see Zhou and Krott, 2016). Even within older children and young adults, the cognitive effects of bilingualism seem to dissipate, and this phenomenon can be related to the finding that the age between 6 and 8 years old is critical for rapid development of executive functioning (Best and Miller, 2010). Often, beneficial effects related to bilingualism are reported in children from birth up to the age of six (e.g., Martin-Rhee and Bialystok, 2008; Kovacs and Mehler, 2009; Morales et al., 2013; Crivello et al., 2016; Woumans et al., 2016), but not in children over the age of six (e.g., Martin-Rhee and Bialystok, 2008; Antón et al., 2014; Abdelgafar and Moawad, 2015), which again is indicative of the transition phase of this age group. Therefore, we compared two groups of younger and older children.

Based on previous studies, we anticipated differences between monolinguals and bilinguals in the younger but not in the older age group. In line with the main focus of this article and our first hypothesis, we expected strategic task tendencies to play a role in the development of the bilingual advantage. If it is true that speed-accuracy trade-offs are one of the reasons why bilingual advantages may be very variable, they should be smaller or non-existent in younger compared to older children.

## Materials and Methods

### Participants

Participants were recruited through schools and after-school-care centers in Belgium. Parents received an information letter on the study’s procedure and filled out an informed consent when they agreed to let their child take part. In total, we obtained authorisations for a large group of 122 children. There were 59 younger children (6-year-olds), of which 29 were monolingual and 30 bilingual. The older children (11-year-olds) consisted of 31 monolinguals and 32 bilinguals. Mean ages and other demographic variables are reported in **Table 1**. With regard to age, younger monolinguals (*M* = 6.7, *SD* = 0.3) did not differ from younger bilinguals (*M* = 6.6, *SD* = 0.3) (*t* < 1.0, *ns*). Older monolinguals (*M* = 11.5, *SD* = 0.3) were slightly younger than older bilinguals (*M* = 11.8, *SD* = 0.5) (*t*_118_ = −2.91, *p* = 0.004), hence we analyzed a subset of these two groups, excluding the two youngest monolinguals and the three oldest bilinguals. This left us with two comparable groups of older monolinguals (*M* = 11.6, *SD* = 0.3) and older bilinguals (*M* = 11.7, *SD* = 0.3) (*t*_56_ = −1.35, *p* = 0.184).

**Table 1:** Demographic data of monolinguals and bilinguals in both age groups.

	Younger children		Older children		Analysis	
	Monolingual	Bilingual	Monolingual	Bilingual	Test	*p*
*N*	29	30	29	29		
Male/female Ratio	17/12	13/17	13/16	11/21	Chi^2^(3) = 2.72	0.437
Age (in years)	6.7 (0.3)	6.6 (0.3)	11.6 (0.3)	11.7 (0.3)	*F*_3,113_ = 2301.71	<0.001
Raven Score	23.7 (3.9)	28.4 (4.4)	24.4 (4.8)	27.9 (3.8)	*F*_3,118_ = 9.30	<0.001
L1 Dutch/French	29/0	30/0	31/0	32/0	–	–
L1 AoA (in years)	0.0 (0.0)	0.0 (0.0)	0.0 (0.0)	0.0 (0.0)	–	–
L1 Proficiency^1^	4.0 (0.0)	3.4 (0.5)	4.0 (0.0)	3.5 (0.5)	*F*_3,113_ = 18.55	<0.001
L2 AoA (in years)	–	0.8 (0.8)	–	0.7 (0.8)	*F*_1,57_ < 1.0	0.618
L2 Proficiecy^1^	–	3.1 (0.9)	–	3.4 (0.6)	*F*_1,57_ = 2.72	0.105
SES^2^	2.6 (0.5)	2.7 (0.4)	2.6 (0.4)	2.5 (0.5)	*F*_3,113_ < 1.0	0.513

*Standard deviations are presented between parentheses. ^1^L1 and L2 proficiency were indicated on a 4-point Likert scale, ranging from 1 (=very low proficiency) to 4 (=very high/native proficiency). ^2^SES was a composite scores of parents’ education levels. Three levels were defined: 1 (=elementary), 2 (=secondary), and 3 (=higher).*

The children’s language background and socioeconomic status (SES) was assessed through a questionnaire. Parents indicated which languages their child had mastered, at which age they acquired them and how proficient they are in them. The parents specified the child’s language proficiency on a 4-point Likert scale, ranging from 1 (=very low) to 4 (=very high/native). They also confirmed that their child did not have any learning disorders, or language development or comprehension issues. SES was a composite score of the parents’ educational levels (elementary, secondary, or higher education) and intelligence was measured through Raven’s Progressive Matrices (Raven, 1938; Raven et al., 1998). **Table 1** shows that monolinguals and bilinguals from both age groups were matched for these measures.

### Design and Procedure

All children were tested individually and the test battery consisted of an intelligence test (Raven’s Matrices) and two control tasks (Simon and flanker). The order of task administration was fixed for all participants: the Simon task came first, followed by the flanker task, to end with the Raven’s test. Testing lasted around 30 min per participant. Breaks were allowed between tasks and between experimental blocks during the control tasks. The children were seated at a distance of approximately 60 cm from the screen. Control task stimuli were presented via Tscope software (Stevens et al., 2006) on an IBM-compatible laptop with 15-inch screen, running XP.

#### Raven’s Progressive Matrices

Raven’s Matrices is a test of analytic reasoning and is considered to be a good measure of fluid intelligence. This test of intelligence was added to our research design because previous research has shown that acquisition of a second language at a young age may foster intellectual development (Woumans et al., 2016). We administered two versions; the colored (Raven et al., 1998) and the standard version (Raven, 1938). The colored matrices are suited for children aged 5 to 11, whereas the standard matrices are suited for age 11 and older. The former test consists of 36 colored drawings with a missing segment which are equally divided over three sets (A, Ab, B) and ordered in terms of increasing difficulty. Participants are asked to complete the drawings indicating one of the six possible answers. A shortened version of the standard matrices was conducted (Van der Elst et al., 2013) to match the amount of items in the colored version, in which only set B, C, and D of the traditional sets A, B, C, D, and E were employed. In set B, each item had six possible options for completion, in set C and D, each item had eight possible options. Since we used subtests instead of the complete one, raw scores were employed as an estimate of participants’ intelligence.

#### Simon Task

A version of the original task by Simon and Rudell (1967) was implemented. Colored dots appeared either on the left or right side of the screen. Participants were asked to press the left (right) key on the keyboard when a green dot appeared, and the right (left) key when the red dot appeared, and this as quickly and as accurately as possible. Response mapping was counterbalanced across participants according to parity of participant number. Each trial began with a fixation of 600 ms, followed by a clear screen and the stimulus, which lasted until the participant’s response or up to 2500 ms. There was a 500 ms blank interval before the next fixation period. The task consisted of 10 randomized practice trials and three blocks of 40 randomized experimental trials. Half of all trials presented the colored dot on the same side of the associated response key (congruent trials) and half on the opposite side (incongruent trials).

#### Flanker Task

A version of the Eriksen flanker task (Eriksen and Eriksen, 1974) was administered, in which five arrows were presented in the center of the screen and participants were asked to indicate the direction (left or right) of the central arrow. The central arrow could either point into the same direction as the four flankers (e.g., < < < < <, congruent trials) or into the other direction (e.g., < < > < <, incongruent trials). Each trial started with a fixation period of 500 ms and was followed by a clear screen and a stimulus presentation of maximum 2500 ms. A blank interval of 500 ms preceded the next trial. The task included 10 practice trials and three blocks of 40 experimental trials each. Half of the trials were incongruent.

## Results

Cognitive control tasks were analyzed by mean reaction times of correct trials (RT) and accuracy scores (ACC) (see **Table 2**). Outlier RTs were trimmed for individual participants by calculating the mean across all trials and excluding any response deviating by more than 2.5 SD of the mean. This procedure eliminated 2.9% of all Simon data and 2.6% of all flanker data. On the Simon task, data from one younger monolingual and one younger bilingual participant were excluded from further analyses due to performance below chance accuracy level of 60%. On the flanker task, data from 10 younger monolingual and 6 younger bilingual participants were excluded from further analyses for the same reason. This exclusion rate is in line with results from previous studies on cognitive control in young children (e.g., Woumans et al., 2017) and can be explained by our choice to administer the default version of the flanker task (thus not the child-friendly version with fish as stimuli) for the purpose of better comparability with the data from the older children. On the remaining data, 2 (Age Group: Younger, Older) × 2 (Language Group: Monolingual, Bilingual) × 2 (Congruency: Congruent, Incongruent) repeated measure ANOVAs were performed to measure the effect of L2 Exposure. Planned comparisons were always employed to disentangle the effects of Age Group and Language Group. When the Levene Statistic was significant, equal variance was not assumed. On the same data, Pearson’s correlational analyses between mean response times and mean accuracy rates were conducted to test for speed-accuracy trade-offs. These analyses were first applied to the entire groups of younger and older bilinguals and then to the bilingual and monolingual groups within these two age groups, separately. Statistical significance was corrected for multiple comparisons using a Bonferroni corrected significance level.

**Table 2:** Reaction times of correct trials (RT – ms) and accuracy scores (ACC – percentages) in the Simon and flanker task split for younger and older monolinguals and bilinguals (standard deviations between parentheses).

	Younger children		Older children	
	Monolingual	Bilingual	Monolingual	Bilingual
*Simon RT*				
Congruent	859 (119)	816 (185)	605 (112)	568 (102)
Incongruent	918 (135)	911 (195)	653 (118)	604 (91)
*Simon ACC*				
Congruent	92.3 (4.7)	89.8 (6.2)	91.4 (7.1)	92.6 (5.1)
Incongruent	88.2 (8.2)	81.8 (9.9)	86.1 (7.8)	88.5 (10.2)
*Flanker RT*				
Congruent	980 (124)	992 (207)	612 (96)	594 (131)
Incongruent	1241 (200)	1241 (240)	757 (137)	684 (159)
*Flanker ACC*				
Congruent	92.1 (6.9)	89.3 (9.3)	97.3 (2.1)	95.1 (4.3)
Incongruent	79.6 (14.0)	70.7 (19.9)	88.7 (6.4)	88.4 (7.5)

### Demographics

Analyses revealed that none of the groups differed for male/female ratio or SES (**Table 1**). There was, however, a difference between younger and older children on Raven scores (*t*_115_ = 27.64, *p* < 0.001), probably due to the fact that raw scores instead of norm scores were used. To our knowledge, no reliable norm scores are available for the subtests that we administered to the participants of the current study (see section “Design and Procedure”). Within the two age groups, none of the Language Groups differed from each other (all *t*s < 1.0, *ns*). Planned comparisons showed that L1 proficiency was, within Age Group, always higher for monolinguals than for bilinguals (Younger: *t*_29_ = 6.16, *p* < 0.001, Older: *t*_28_ = 4.53, *p* < 0.001). Independent samples showed that, across Age Groups, there were no differences between monolinguals and bilinguals on L2 AoA (*t*_57_ < 1.0, *p* = 0.618) and self-reported L2 proficiency (*t*_57_ = −1.65, *p* = 0.105).

### Simon Task

Descriptive statistics are summarized in **Table 2**. In the RT analysis, the main effect of Congruency was significant (*F*_1,111_ = 147.66, *p* < 0.001, $\eta_{p}^{2}$ = 0.571), indicating faster responses to congruent trials (*M* = 711 ms, *SD* = 184) than to incongruent trials (*M* = 770 ms, *SD* = 200). There was also a main effect of Age Group (*F*_1,111_ = 114.66, *p* < 0.001, $\eta_{p}^{2}$ = 0.508) with faster RTs for older children, but no main effect of Language Group (*F*_1,111_ = 1.87, *p* = 0.174, $\eta_{p}^{2}$ = 0.017). The two-way interaction between Congruency and Age Group was significant (*F*_1,111_ = 12.32, *p* = 0.001, $\eta_{p}^{2}$ = 0.100), revealing a smaller Simon effect for older children (*M* = 42 ms, *SD* = 40) than for younger children (*M* = 77 ms, *SD* = 64). The interaction between Congruency and Language Group was not significant (*F*_1,111_ = 1.39, *p* = 0.240, $\eta_{p}^{2}$ = 0.012), and neither was the one between Age Group and Language Group (*F*_1,111_ < 1.0, *ns*). Yet, further analyses disclosed a significant three-way interaction between Congruency, Language Group, and Age Group (*F*_1,111_ = 6.05, *p* = 0.015, $\eta_{p}^{2}$ = 0.052). Planned comparisons demonstrated a significant difference on the Simon effect for younger monolinguals and bilinguals (*t*_54.25_ = −2.16, *p* = 0.036), with monolinguals displaying a smaller effect, and no significant difference between the older language groups (*t*_55.64_ = 1.18, *p* = 0.245).

In the accuracy analyses, there was a main effect of Congruency (*F*_1,111_ = 49.68, *p* < 0.001, $\eta_{p}^{2}$ = 0.309), with higher scores for congruent trials (*M* = 91.5%, *SD* = 5.9) than for incongruent trials (*M* = 86.1%, *SD* = 9.4). There was no effect of Age Group (*F*_1,111_ = 1.90, *p* = 0.171, $\eta_{p}^{2}$ = 0.017) or Language Group (*F*_1,111_ = 1.204, *p* = 0.275, $\eta_{p}^{2}$ = 0.011). There was an Age Group^∗^Language Group interaction (*F*_1,111_ = 3.48, *p* = 0.011, $\eta_{p}^{2}$ = 0.056). The difference between younger monolinguals and bilinguals (4.43%) was larger than that between older monolinguals and bilinguals (1.77%). None of the other interactions were significant either (all *p*s > 0.095).

**FIGURE 1:**
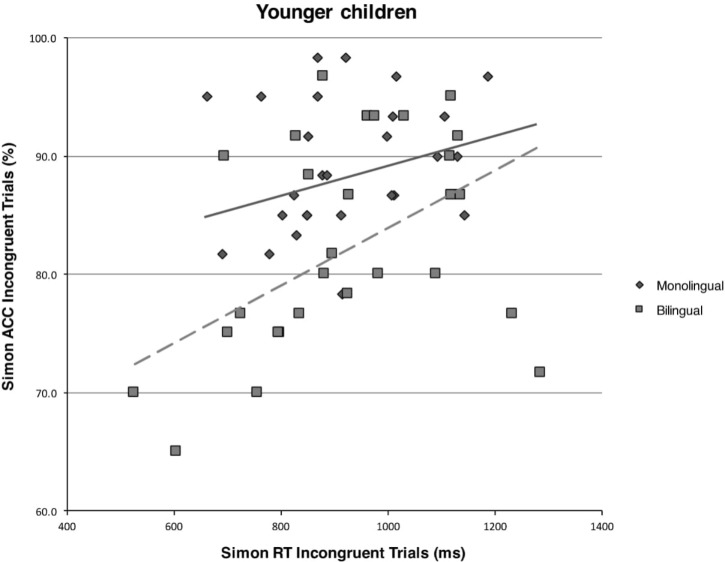
Scatterplot and regression fit line showing the relationship between mean response times (in milliseconds) and mean accuracy rates (in percentages) on incongruent trials of the Simon task for the monolingual and bilingual younger children.

A Pearson’s correlational analysis on the subset of younger monolingual children revealed no significant speed-accuracy trade-off on any of the investigated measures, all *p*s > 0.017, the Bonferroni corrected significance level. The one on the subset of younger bilingual children, however, indicated a highly significant speed-accuracy trade-off for incongruent trials (*r*_29_ = 0.48, *p* = 0.001) but not for congruent trials or global performance (all *p*s > 0.017). See **Figure 1** for a graphical representation of the comparison between younger bilingual and monolingual children on the correlation between accuracy rates and response times on incongruent trials of the Simon task.

**FIGURE 2:**
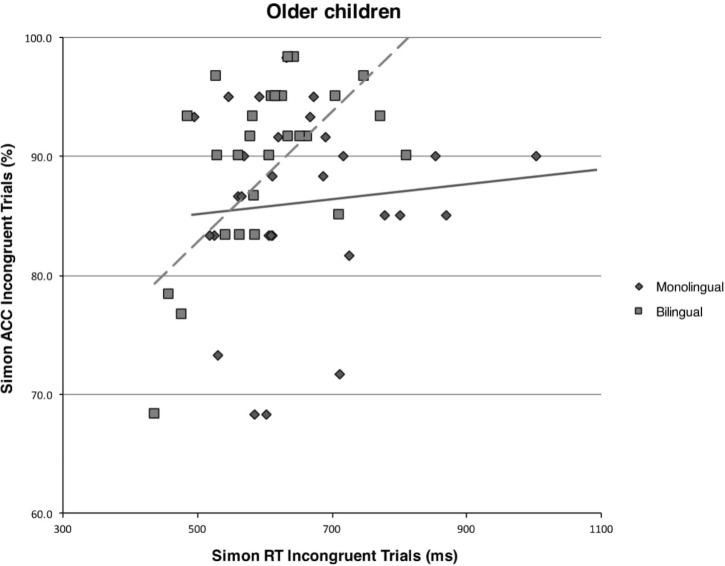
Scatterplot and regression fit line showing the relationship between mean response times (in milliseconds) and mean accuracy rates (in percentages) on incongruent trials of the Simon task for the monolingual and bilingual older children.

The same analyses on the subset of older monolingual children also disclosed no significant results (all *p*s > 0.05). In contrast, analyses on the subset of older bilingual children showed a highly significant speed-accuracy trade-off for global performance (*r*_29_ = 0.53, *p* = 0.003), and for incongruent (*r*_29_ = 0.49, *p* = 0.007) but not congruent trials (*r*_29_ = 0.15, *p* = 0.435). See **Figure 2** for a graphical representation of the comparison between older bilingual and monolingual children on the correlation between accuracy rates and response times on incongruent trials of the Simon task.

### Flanker Task

Descriptive statistics are summarized in **Table 2**. For RTs, the main effect of Congruency was significant (*F*_1,97_ = 280.44, *p* < 0.001, $\eta_{p}^{2}$ = 0.743), indicating faster responses to congruent trials. There was also a main effect of Age Group (*F*_1,97_ = 206.74, *p* < 0.001, $\eta_{p}^{2}$ = 0.681), demonstrating faster RTs for older children, but no effect of Language Group (*F*_1,97_ < 1.0, *p* ns.). There was, however, a Congruency^∗^Age Group interaction (*F*_1,97_ = 38.19, *p* < 0.001, $\eta_{p}^{2}$ = 0.282), with a smaller flanker effect for older children (*M* = 118 ms, *SD* = 68) than for younger children (*M* = 255 ms, *SD* = 152). Although repeated measures analyses exposed no other two-way interaction effects and no three-way interaction between Congruency, Language Group, and Age Group (*F*_1,97_ < 1.0, *p* ns.), planned comparisons still signaled a significant difference between older monolinguals and bilinguals on the flanker effect (*t*_55.96_ = 3.40, *p* = 0.001), with a smaller effect for bilinguals (*M* = 90 ms, *SD* = 63) as opposed to monolinguals (*M* = 145 ms, *SD* = 61).

Measuring accuracy, similar results were obtained, with higher scores for congruent trials (*F*_1,97_ = 92.07, *p* < 0.001, $\eta_{p}^{2}$ = 0.487) and for older participants (*F*_1,97_ = 35.99, *p* < 0.001, $\eta_{p}^{2}$ = 0.271), and for monolinguals (*F*_1,97_ = 5.06, *p* < 0.05). There was also a Congruency^∗^Age Group interaction (*F*_1,97_ = 10.75, *p* = 0.001, $\eta_{p}^{2}$ = 0.100), with older children (*M* = 7.6%, *SD* = 5.9) having a smaller accuracy effect than younger children (*M* = 15.5%, *SD* = 27.3). No other effects were significant.

**FIGURE 3:**
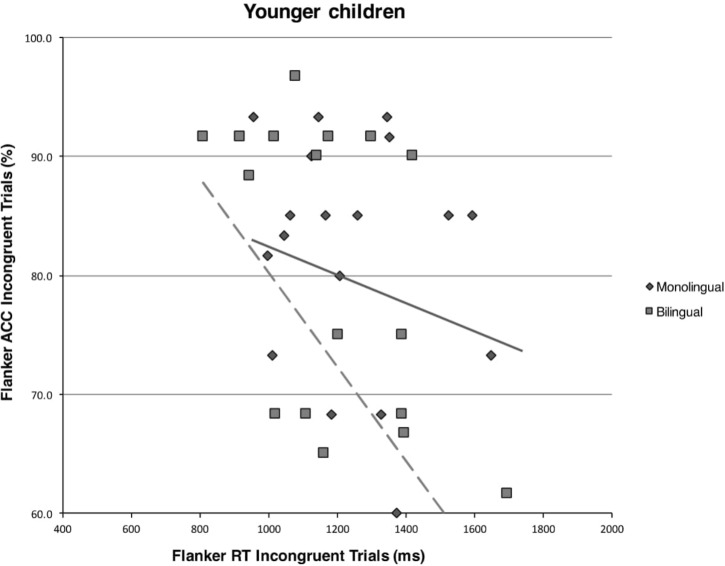
Scatterplot and regression fit line showing the relationship between mean response times (in milliseconds) and mean accuracy rates (in percentages) on incongruent trials of the flanker task for the monolingual and bilingual younger children.

Pearson’s correlational analyses on the subset of younger monolingual or young bilingual children did not reveal any significant speed-accuracy trade-offs (all *p*s > 0.017, the Bonferroni corrected significance level). See **Figure 3** for a graphical representation of the comparison between younger bilingual and monolingual children on the correlation between accuracy rates and response times on incongruent trials of the flanker task.

**FIGURE 4:**
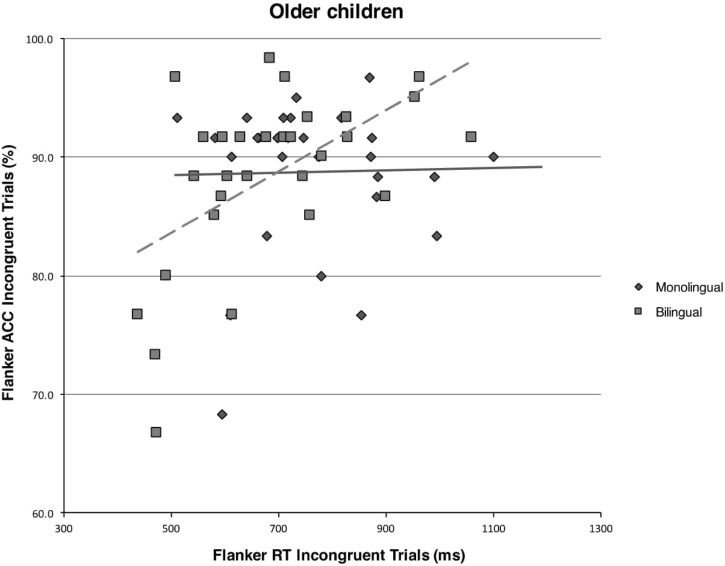
Scatterplot and regression fit line showing the relationship between mean response times (in milliseconds) and mean accuracy rates (in percentages) on incongruent trials of the flanker task for the monolingual and bilingual older children.

A Pearson’s correlational analysis on the subset of older monolingual children revealed no significant correlations at all (all *p*s > 0.017). The same analysis on the subset of older bilingual children, however, revealed highly significant speed-accuracy trade-off for global performance (*r*_29_ = 0.54, *p* = 0.002) and for incongruent trials (*r*_29_ = 0.55, *p* = 0.002), but not for congruent trials (*p*s > 0.017). See **Figure 4** for a graphical representation of the comparison between older bilingual and monolingual children on the correlation between accuracy rates and response times on incongruent trials of the flanker task.

## Discussion

The aim of this study was to investigate the role of cognitive development and speed-accuracy trade-offs in the bilingual advantage controversy. Therefore, two groups of children (monolinguals and bilinguals) from two different age categories (younger and older children) were tested on cognitive control performance in two of the most frequently used tasks in the bilingualism literature: the Simon task and the flanker task. In line with previous findings, we only expected group differences between bilinguals and monolinguals in the youngest age group but not in the older one (Bialystok et al., 2004). Nevertheless, we did not merely intend to compare bilinguals to monolinguals in a between-group design, but also determine whether the absence or presence of differences in cognitive control are related to strategic task tendencies (i.e., optimizing either speed or accuracy performance) to resolve conflict. Our expectation was that bilinguals would follow a particular strategy to carry out these tasks, as indicated by a significant speed-accuracy trade-off, while monolinguals would show a more random pattern of behavior. Most crucially, we anticipated a relationship between speed-accuracy trade-off and the bilingual advantage, in the sense that such a trade-off could hide potential group differences.

### No Clear-Cut Evidence for a Bilingual Advantage

A first important finding of this study was that there was no clear-cut evidence for a bilingual advantage. On the one hand, we did observe a smaller congruency effect for the older bilinguals on the flanker task; whereas, on the other, we found smaller congruency effects for younger monolinguals on the Simon task and higher accuracy scores for monolinguals in general on the flanker. We could therefore not confirm our first hypothesis that the bilingual advantage would only be found in the youngest and not the oldest group. Our results are, however, in line with recent meta-analyses on the bilingual advantage showing dubious results (de Bruin et al., 2014; Lehtonen et al., 2018). Furthermore, because both global measures of cognitive control (performance on the task as a whole, see, for instance, Costa et al., 2009) and specific measures (performance on incongruent trials only, see, for instance, Marzecova et al., 2013) were not consistently affected by bilingualism, we were unable to distinguish between interpretations of the bilingual advantage in terms of monitoring or inhibition.

### Speed-Accuracy Trade-Offs

The major interest of the current study did not lie in the quest for a bilingual advantage, but rather in the investigation of potential differences between bilinguals and monolinguals in strategic task tendencies. In line with our expectations, we found evidence for speed-accuracy trade-offs only for bilinguals and not monolinguals, and this in the two tasks under scrutiny. These results reveal for the first time a group difference in the strategies underlying the execution of cognitive control tasks. Confronted with the need for conflict resolution in a control task, bilinguals sought to optimize their performance by choosing a clear strategy, either by boosting their response times at the cost of accuracy, or by improving their accuracy rate by slowing down their performance. The monolinguals did not implement a similar strategy, as their performance did not show any relationship between speed and accuracy. We suggest that the cause for this between-group difference is comparable to that of the bilingual advantage, as it may also constitute the combination of training and transfer effects. Bilinguals face the constant need for conflict resolution as they have to manage two language systems, either when they activate the target language in face of interference from the non-target language, or when they switch between languages (e.g., Moreno et al., 2010; Tse and Altarriba, 2012). Compared to other language users, it has been found that bilinguals develop specific strategies to solve these linguistic conflicts (e.g., FrenckMestre and Pynte, 1997; Blanco-Elorrieta and Pylkkanen, 2016), and in the domain of language contact at the level of the individual language user, these have been labeled as ‘bilingual optimisation strategies’ (Muysken, 2013; Indefrey et al., 2017). In the same vein, speed-accuracy trade-offs can be seen as an optimisation strategy intended to boost performance in conflict situations. Interestingly, the implementation of this strategy in bilinguals in the Simon task was only visible for incongruent trials, or those trials for which conflict resolution is needed to attend to the task-relevant dimension in face of competition from a task-irrelevant dimension.

These findings suggest that the optimisation strategies that bilinguals develop when dealing with linguistic conflict may transfer into the non-verbal domain and that they may apply to any situation where a bilingual individual encounters conflict. As such, this training and transfer effect is an elaboration of the theoretical foundations of the bilingual advantage in cognitive control (see Kroll and Bialystok, 2013) as it suggests that a crucial difference between bilinguals and monolinguals regarding cognitive control lies in the strategies bilinguals actively recruit to resolve conflict, even when their response times or accuracy rates do not significantly deviate from those of monolinguals. This observation may have important implications for the bilingual advantage debate. Previously, the quest for bilingual effects in cognitive control was confined to an investigation of potential differences in the speed (or accuracy) of processing, and the absence of these differences led to the assumption that there is no consistent evidence for a bilingual advantage (Paap and Greenberg, 2013; Paap et al., 2014; von Bastian et al., 2016). However, this quest for behavioral advantages could interfere with the different strategies used by bilinguals and monolinguals to carry out these tasks. If bilinguals seek – even unconsciously – to optimize their performance, only one of these two dimensions will be positively affected. Between-group differences in speed-accuracy trade-offs could thus explain why bilingual advantages are observed either in terms of processing speed or accuracy (compare to the studies listed by Paap et al., 2014).

We also propose that differences in strategic task tendencies may mask potential group differences in accuracy or speed. In spite of the between-group differences in speed-accuracy trade-offs, no similar differences were detected when speed and accuracy were analyzed separately. However, our descriptive statistics revealed a tendency of lower response times for the bilinguals and higher accuracy for the monolinguals. In one subgroup (the older children on the flanker task), this even led to a monolingual advantage in accuracy. Within the explanatory framework of strategy choices, we suggest that this is the result of the bilinguals’ optimisation strategy to boost response times at cost of lower accuracy. The question may arise why these group differences in speed-accuracy trade-offs have led on only one occasion to group differences in speed or accuracy. One reason for this could be that while the bilinguals as a group make use of optimisation strategies to resolve conflict in control tasks, the choice for a speed or an accuracy strategy may differ between individuals based on their need for interference suppression in daily bilingual language use related to variables such as the differences in proficiency level between L1 and L2, the degree of language switching, and the typological distance between both languages. Only if most or nearly all bilingual participants implement the same strategy to resolve conflict, a clear advantage may be found on that dimension. Previous studies seem to suggest that advantages are more frequently observed in speed than in accuracy, which may reveal a preference for a speed strategy among bilinguals (compare to the studies listed by Paap et al., 2014). However, the design of the current study did not allow us to make any claims on this issue and this is also one of its limitations. We therefore strongly recommend future studies on the bilingual to manipulate the speed and accuracy strategy by explicitly instructing which dimension must be prioritized (Wylie et al., 2009; Uemura et al., 2013). In line with the interpretation of this study’s findings, we expect bilinguals to benefit more from these explicit instructions because they have been trained in the usage of optimisation strategies.

### Development

The final research question of the current study dealt with the developmental aspects of the bilingual advantage and the potentially interfering role of speed-accuracy trade-offs in the manifestation of this advantage. Compatible with the results for the test population as a whole, an age difference was found between the flanker and the Simon task specifically for the bilingual subgroup. Whereas speed-accuracy trade-offs were observed in both age groups for the Simon task, only the older children showed a correlation between speed and accuracy on the flanker task. These findings were – at least for the Simon task – not in line with our own expectations, as we anticipated a speed-accuracy trade-off in the older but not in the younger children.

A first reason for this may be related to the specific characteristics of each of the two cognitive control tasks, which do not only differ from each other in the mean length of response times (which is significantly higher for the flanker than for the Simon task), but also in the underlying mechanisms of conflict resolution due to compatibility or congruency between stimulus and response (Kornblum et al., 1990). On an incongruent flanker trial, one (task-relevant) dimension of the stimulus (the direction of the central arrow) conflicts with another (but task-irrelevant) dimension of the same stimulus (the direction of the surrounding arrows). On the other hand, on an incongruent Simon trial, a (task-relevant) dimension of the stimulus (the color of the square) conflicts with a (task-irrelevant) dimension of the response (the location of the response). As a result of these differences, both types of conflict are processed independently (Li et al., 2014) with stimulus–stimulus conflicts (as generated in a flanker task) inducing stronger behavioral effects (Fruhholz et al., 2011) than stimulus–response conflicts (as generated in a Simon task). As it may be more effortful to process a task that induces stronger behavioral effects, it could be that only older children have the ability to make strategic choices on stimulus–stimulus conflicts in the flanker task, whereas the same does not apply to the easier stimulus–response conflicts in the Simon task.

The second reason for the mismatch between the current study’s hypotheses and its actual findings is that our expectations regarding the role of development were related to an anticipated bilingual advantage in the younger but not in the older children. As we did not consistently observe such an advantage, the rationale behind developmental differences in speed-accuracy trade-off was no longer present. We therefore assume that the developmental differences between the two tasks were solely caused by the characteristics of the individual tasks instead of any possible relationship with a bilingual advantage.

## Conclusion

The most important contribution of the current study to the expanding bilingual advantage literature is that cognitive control differences between bilinguals and monolinguals can manifest themselves in strategic task tendencies implemented to resolve conflict, even when consistent performance differences between bilinguals and monolinguals in terms of speed and accuracy are absent. The crucial difference between our two language groups was that only bilingual children showed a consistent pattern of speed-accuracy trade-offs on the flanker and Simon task. Comparable to the theoretical foundations of the bilingual advantage, we have related these differences to a combined training and transfer effect as a result of the specific demands of bilingual language usage. Our findings prompt a nuanced view on the bilingual advantage debate: as we did not find any evidence for performance differences, the term ‘advantage’ may be a misnomer for what is happening in the bilingual mind (as compared to monolinguals); but at the same time, the variation in implemented strategies to resolve conflict illustrate the impact that constant exposure and usage of two (or more) language systems may have on cognitive processing in the bilingual mind (compare to Woumans et al., 2016).

## Ethics Statement

This study was carried out in accordance with the recommendations of the Ghent University’s Faculty of Psychology’s ethical guidelines, with written informed consent from all subjects. All subjects gave written informed consent in accordance with the Declaration of Helsinki. The protocol was approved by the Ethical Committee of the Faculty of Psychology at Ghent University.

## Author Contributions

ES and EW determined the research questions and performed the statistical analysis. EW programmed the tasks and conducted the experiments. WD made suggestions. ES drafted the manuscript. EW and WD provided critical assessments.

## Conflict of Interest Statement

The authors declare that the research was conducted in the absence of any commercial or financial relationships that could be construed as a potential conflict of interest.
